# Marine Compounds and Cancer: Updates 2020

**DOI:** 10.3390/md18120643

**Published:** 2020-12-15

**Authors:** Sergey A. Dyshlovoy, Friedemann Honecker

**Affiliations:** 1 Laboratory of Pharmacology, A.V. Zhirmunsky National Scientific Center of Marine Biology, Far Eastern Branch, Russian Academy of Sciences, 690041 Vladivostok, Russia; 2 Laboratory of Experimental Oncology, Department of Oncology, Hematology and Bone Marrow Transplantation with Section Pneumology, Hubertus Wald-Tumorzentrum, University Medical Center Hamburg-Eppendorf, 20251 Hamburg, Germany; Friedemann.Honecker@zetup.ch; 3 Martini-Klinik, Prostate Cancer Center, University Hospital Hamburg-Eppendorf, 20251 Hamburg, Germany; 4 Tumor and Breast Center ZeTuP St. Gallen, 9000 St. Gallen, Switzerland

By the end of the year 2020, there are nine marine-derived anticancer drugs available on the market, and the field is currently growing exponentially. This process is stipulated by improvements in the development of biomedical sciences in general and recent approval of new and exciting anticancer medications in particular, which were developed based on small molecules of marine origin.

Looking back, it is noteworthy that at the very beginning of 2018, when we published an article on updates in the field of marine anticancer agents, there were only four marine-derived drugs approved for the treatment of cancer and cancer-related conditions [1]. Those were **cytarabine** (Cytosar-U^®^, the very first marine-derived drug [2] approved in 1969 produced by Pfizer [3]), **trabectedin** (Yondelis^®^, produced by PharmaMar), **eribulin mesylate** (Halaven^®^, produced by Eisai Inc.), and the antibody–drug conjugate (ADC) **brentuximab vedotin** (Adcetris^®^, produced by Seattle Genetics) [1]. Within only three years since 2018, five (!) new drugs have been approved for the treatment of different cancer types all over the world; two of them have been approved only recently in 2020. Thus, to the four “marine” pharmaceuticals listed above, the following medications were added:

- **Plitidepsin** (Aplidin^®^, produced by PharmaMar), dehydrodidemnin B, is an ascidian depsipeptide binding to eEF1A2 and inducing an oxidative stress in cancer cells; the drug was first approved in 2018 in Australia for the treatment of multiple myeloma, leukemia, and lymphoma [4].
- **Polatuzumab vedotin** (Polivy^TM^, produced by Genentech, Roche) is an ADC that consists of MMAE (monomethyl auristatin E, an analogue of dolastatin 10, which is a peptide toxin of symbiotic marine cyanobacteria) conjugated with the CD76b-specific monoclonal antibody polatuzumab. The antibody provides a specific delivery of MMAE to cancer cells, where following the proteolytic ADC cleavage and release of the “warhead” molecule (MMAE), an inhibition of tubulin polymerization leading to the cancer cells’ death can be achieved. The drug was approved by the FDA in 2019 for the treatment of B-cell lymphomas, non-Hodgkin lymphomas, and chronic lymphocytic leukemia [5].
- **Enfortumab vedotin** (PADCEV^TM^, produced by Astellas Pharma and Seattle Genetics) is another ADC consisting of MMAE (see above) and an antibody specific to nectin-4. It was approved in 2019 for the treatment of metastatic urothelial cancer [6].
- **Belantamab mafodotin** (Blenrep^TM^, produced by GlaxoSmithKline) is yet another ADC consisting of MMAF (monomethyl auristatin F, one of the MMAE derivatives) as the warhead, bound to an antibody targeting BCMA (B-cell maturation antigen). Similar to MMAE, MMAF targets tubulin polymerization. The drug was approved in 2020 for the treatment of relapsed and refractory multiple myeloma [7].
- **Lurbinectedin** (Zepzelca^TM^, produced by PharmaMar) is a synthetic derivative of trabectedin (see above) that binds to the minor groove of DNA and exerts its anticancer action via inhibition and degradation of RNA polymerase II. The drug was approved in 2020 for the treatment of metastatic small cell lung cancer [8].

According to the Marine Pharmacology web page provided by Prof. Alejandro M. S. Mayer and colleagues (https://www.midwestern.edu/departments/marinepharmacology.xml), there are currently another 23 “marine” molecules in different stages of clinical development in various cancer entities [9]. The vast majority of these drug candidates (i.e., 19 out of 23 (83%)) are being tested as anticancer drugs. It should, however, be noted that most of the molecules (70%) either are ADC derivatives of MMAE or MMAF or are already approved drugs undergoing trials in entities where they have not yet been approved (e.g., lurbinectedin in ovarian, breast, and small cell lung cancer) [9].

To keep track in this dynamic area and also to offer a platform on research dealing with new and promising marine compounds possessing anticancer activity, we started a topical collection, “Marine Compounds and Cancer” (http://www.mdpi.com/journal/marinedrugs/special_issues/marine-compounds-cancer), in 2015 [10]. This topical collection covers the whole scope of agents showing in vitro and in vivo anticancer properties, which are able to prevent cancer development or can kill existing cancer cells. We publish data on both novel and previously characterized compounds, which either just have started to come to the attention of biomedical scientists or already have become established drugs [1,10,11].

A number of articles have been published in the topical collection before 2018 [1,10]. Since then, many new high-quality manuscripts have been submitted and subsequently published. In total, 6 review and 24 original research articles have been accepted in this topical collection.

In the following, we will briefly review the data presented in those publications. Li and colleagues reviewed the recent data on chemopreventive, antineoplastic, chemosensitive, procoagulant, and anticoagulant activities of **sepia ink polysaccharide** [12]. Ćetković et al. summarized findings on **cancer-related genes and proteins** found in marine sponges and provided insight into sponge genome and proteome [13]. Fan et al. reviewed **marine-derived compounds that have been described to be active in human prostate cancer models** both in vitro and in vivo. Molecules with activity in this entity belong to different molecular classes, such as nucleotides, amides, quinones, polyethers, and peptides, and possess different anticancer-related activities, such as antioxidant, antiangiogenetic, antiproliferative, and apoptosis-inducing activities [14]. A nice concise review by Martínez Andrade and coauthors covers the topic of **marine microalgae and their unique molecules** that have shown anticancer properties [15]. Van Andel et al. outlined different **chromatographic bioanalytical methods that are used for the quantitative determination of marine-derived molecules**, which have shown anticancer properties [16]. Ha et al. compiled recent findings on the design, synthesis, and biological activity of so-called **hybrid (chimera) molecules, which are based on marine natural compounds** and their derivatives [17].

In the 24 research articles published in the topical collection since the beginning of 2018, data from a number of both new and previously known marine-derived compounds have been reported. Guo et al. synthesized a novel bromophenol derivative (**BOS-102**), which is active in a human lung cancer cell model. The underlying mechanism of action could be identified as an induction of apoptosis and cell cycle arrest via ROS-mediated PI3K/Akt and MAPK signaling [18]. Aldairi and colleagues described **glycosaminoglycan-like polysaccharides** isolated from the marine mollusk *Cerastoderma edule.* This compound has exhibited antiproliferative activity in chronic myeloid leukemia as well as in relapsed acute lymphoblastic leukemia models [19]. Manh Hung et al. studied the effect of **gliotoxin** in combination with adriamycin on non-small cell lung cancer cells resistant to Adriamycin. The authors showed that gliotoxin can induce an intrinsic apoptotic response in cancer cells and activate the p53 protein. Additionally, gliotoxin enhanced the cytotoxic effect of Adriamycin [20]. Loret et al. isolated and characterized the small protein **BDS-5** from the sea anemone *Anemonia viridis*, which shows similarities to Kunitz-type inhibitors. BDS-5 has shown to possess antiangiogenic activity, which may be exploited in anticancer therapy [21]. Using a pheochromocytoma model, Bechmann and coauthors showed anticarcinogenic and antimetastatic activities of **aeroplysinin-1 and isofistularin-3**, compounds that were previously isolated from the marine sponge *Aplysina aerophoba*. Additionally, aeroplysinin-1 downregulated integrin β1 [22]. Hao and colleagues studied the anticancer activity of **phycocyanin** in non-small cell lung cancer cells. The authors reported that phycocyanin can regulate NF-κB signaling and induce apoptosis and cell cycle arrest, and suppresses cell migration, proliferation, and colony formation [23]. An article by Lin et al. describes an anticancer effect of the sponge-derived pentacyclic alkaloid **manzamine A** in colorectal cancer cells. In their research, using a microarray-based gene expression analysis, the authors revealed an effect of the treatment on caspase-dependent intrinsic apoptosis, DNA repair executed via an inhibition of CDKs p53/p21/p27 cell cycle arrest, and mRNA metabolism [24]. Rath et al. reported an anticancer effect of the well-known spongian alkaloid **fascaplysin** in lung cancer cells and circulating tumor cells from lung cancer. Fascaplysin induced ATM signaling, initiated by treatment-induced DNA damage, and increased the anticancer action of cisplatin [25]. Zhu and colleagues showed the antiangiogenic activity of **rLm-cystatin F**, a homologue of cystatin F, which was isolated from the buccal glands of *Lampetra morii*. rLm-cystatin F can suppress the migration, invasion, adhesion, and tube formation of HUVEC cells [26]. Ting and Chen demonstrated the anticancer activity of the antimicrobial peptide **tilapia piscidin 4** (TP4) derived from the fish species *Nile tilapia* in non-small cell lung cancer cells. TP4 induced necrotic death of cancer cells and increased the effect of the EGFR inhibitors erlotinib and gefitinib in EGFR-mutated non-small cell lung cancer cells [27]. Liang and colleagues utilized a fragment-based drug design in order to optimize the structure of the spongian brominated tyrosine **itampolin A**, which has previously been reported to be a potent p38α inhibitor. The authors synthesized and selected the most potent derivative, which showed activity in non-small cell lung cancer cells [28]. Qiao et al. investigated the anticancer activity of **tetracenomycin X** in human lung cancer cells in vitro and in vivo. They showed that the compound induces cell cycle arrest via both direct induction of cyclin D1 degradation by the proteasomal system and indirect downregulation of cyclin D1 due to the activation of p38 and c-JUN [29]. Groult and colleagues showed that algal **λ-carrageenan oligosaccharides** inhibit the migration of MDA-MB-231 breast cancer cells [30]. Xu et al. reported new and previously known natural dimeric naphthopyrones that are cytotoxic in several human cancer cells lines and could demonstrate that this cytotoxicity is executed via ROS-mediated apoptosis. Additionally, the authors showed that the PI3K/Akt pathway also plays a role in inducing this cytotoxic effect [31]. Zhou et al. reported on the activity of four new ansamycins, namely, **divergolides T–W**, and two previously known individual compounds isolated from the culture of mangrove-derived actinomycete *Streptomyces* sp. KFD18. Some of these compounds exhibited potent cytotoxicity in several human cancer cell lines, executed via the apoptotic pathway [32]. Lin and colleagues reported a suppressive effect of **actinomycin V** on the migration and invasion of human breast cancer cells. This effect can be explained by the inhibition of the Snail/Slug-mediated EMT (epithelial–mesenchymal transition) under drug treatment [33]. Teruya et al. analyzed the activity of a low **molecular weight fucoidan**. They identified that it can suppress the growth of fibrosarcoma cancer cells without having an effect on the proliferation of noncancer TIG-1 cells. The underlying mechanism of this activity has been identified as a specific inhibition of the PD-L1/PD-L2 expression in cancer cells [34]. An analysis of the **αO-conotoxin GeXIVA** activity in a breast cancer cell model performed by Sun and colleagues revealed the antiproliferative activity of this conotoxin. This effect can be explained by the downregulating α9-nAChR (α9 nicotine acetylcholine receptor), ultimately leading to cell cycle arrest [35]. Kapustina et al. reported the isolation of four new humulane sesquiterpenoids, **leptogorgins A–C** and a new dihydroxyketosteroid, **leptogorgoid A**. Some of these compounds exhibited cytotoxicity and selectivity in human drug-resistant prostate cancer cells in vitro [36]. Zhou et al. described the proapoptotic activity of the previously known spongian scalarane sesterterpenoid **12-deacetyl-12-epi-scalaradial** in human cancer HeLa cells. The authors postulate that this cytotoxic effect is executed via the MAPK/ERK pathway, as well as by the activation of the Nur77 nuclear receptor activity [37]. Capasso et al. showed an antiproliferative activity and selectivity of **mycalin A** and its derivatives, synthesized by the same group, in melanoma and cervical cancer models [38]. Shubina and colleagues reported the discovery of a new structural group of spongian monosulfated polyoxygenated steroids named gracilosulfates. In their work, they isolated seven **gracilosulfates, A–G**. These molecules can inhibit the expression of PSA in human prostate cancer cells, thereby inducing an anticancer effect [39]. Finally, Delgado-Roche et al. reported that metabolites of *Thalassia testudinum*, in particular **thalassiolin B**, exhibit chemopreventive and antigenotoxic activity, which can be of use in anticancer therapy. This effect was at least partially mediated by the inhibition of the CYP1A1-mediated benzo[a]pyrene-induced transformation (i.e., by suppressing the effects of oxidative and mutagenic stress) [40]. 

The scientific and medical community embraces new biologically active compounds, which will hopefully be further developed into clinically useful drugs. Putting marine-derived molecules in the focus of research on natural products and medical chemistry has already resulted in the development of several effective drugs that have saved thousands of lives. Therefore, we thank all the authors who have contributed to this important field and have added to the topical collection “Marine Compounds and Cancer” of *Marine Drugs*! 
